# The evolving role of radiation in pancreatic cancer

**DOI:** 10.3389/fonc.2022.1060885

**Published:** 2023-01-11

**Authors:** Midhun Malla, Fatemeh Fekrmandi, Nadia Malik, Hassan Hatoum, Sagila George, Richard M. Goldberg, Sarbajit Mukherjee

**Affiliations:** ^1^ West Virginia University Cancer Institute, Morgantown, WV, United States; ^2^ Department of Radiation Medicine, Roswell Park Comprehensive Cancer Center, Buffalo, NY, United States; ^3^ Hematology/Oncology, Stephenson Cancer Center, University of Oklahoma Health Sciences Center, Oklahoma City, OK, United States; ^4^ Department of Medicine, GI Medical Oncology, Roswell Park Comprehensive Cancer Center, Buffalo, NY, United States

**Keywords:** pancreatic cancer, chemo - radiotherapy, radiation, resectability, radiation techniques

## Abstract

Pancreatic cancer is the fourth leading cause of cancer mortality in the United States. Chemotherapy in resectable pancreatic cancer has improved survival by 10-20%. It only converted 10-30% of the borderline resectable and locally advanced pancreatic cancers to be surgically resectable. Radiation therapy has a documented role in managing localized pancreatic cancer, more so for borderline and locally advanced pancreatic cancer, where it can potentially improve the resectability rate of a given neoadjuvant treatment. The role of radiation therapy in resected pancreatic cancer is controversial, but it is used routinely to treat positive margins after pancreatic cancer surgery. Radiation therapy paradigms continue to evolve with advancements in treatment modalities, delivery techniques, and combination approaches. Despite the advances, there continues to be a controversy on the role of radiation therapy in managing this disease. In this review article, we discuss the recent updates, delivery techniques, and motion management in radiation therapy and dissect the applicability of this therapy in pancreatic cancer.

## 1 Introduction

Radiation therapy (RT) has been utilized in managing patients with pancreatic cancer in the neoadjuvant, adjuvant, locally recurrent, and metastatic settings. Radiation therapy paradigms continue to evolve with advancements in treatment modalities, delivery techniques, and combination approaches. Despite the advances, there continues to be a debate about the benefits of radiation therapy in managing this disease.

Approximately 50% of patients with resectable pancreatic cancer cannot receive adjuvant therapy because of surgical complications, delayed recovery, and early recurrence. This has encouraged the widespread adoption of neoadjuvant therapeutic strategies in patients with resectable or borderline resectable pancreatic cancer. With this shift in the treatment paradigm, the indications and need for the inclusion of radiation therapy in the neoadjuvant setting continue to be a topic of intense debate.

Pancreatic cancer modifies the immune, vascular, and connective tissue components around the tumor, creating a favorable tumor microenvironment (TME) for its growth and immune evasion (1). The goal of RT delivery in a neoadjuvant setting is multi-fold; to accomplish a margin-negative resection and to minimize the risk of local recurrence while limiting RT exposure to surrounding organs (2). These goals should be balanced with the adverse effect profile. Whether the incorporation of radiation in neoadjuvant therapy translates into improved survival outcomes is yet to be seen. Indeed, most trials in the adjuvant setting have not demonstrated survival benefits, and some studies reported detrimental effects from RT.

RT uses high-energy photons, electrons, protons, and other sources to cause DNA damage in tumor cells leading to lethality. Ionizing radiation creates ion pairs in water that could cause base damage, single strand, or double-strand breaks in proximity to DNA. Faulty tumor cell repair mechanisms could lead to genetic instability, cell death, and senescence (3). Notably, not all cells are universally responsive to radiation, as the mode of death may depend on the dose and cell type (4). Conversely, pancreatic cancer cells demonstrate intrinsic and acquired modes of radioresistance through different mechanisms. Radiosensitizers, including known and novel agents, are under investigation in pre-clinical and clinical studies (5).

Delivery of higher biologically equivalent doses in shorter treatment periods through highly conformal stereotactic body radiation therapy (SBRT) with reduced treatment-related toxicities has attracted much attention. Data suggesting the benefit of elective nodal irradiation and potential sites of microscopic spread is also accumulating (6, 7).

Radiation toxicity in normal tissues could manifest as acute side effects, including fatigue, skin irritation, nausea/vomiting, anorexia, weight loss, and stomach or duodenal ulcers. It could also manifest as late radiation side effects, including chronic fatigue, skin discoloration, stomach or duodenal ulcers, bowel obstruction, and liver or kidney dysfunction. These toxicities are tracked and quantified through different methods, including Common Toxicity Classification for Adverse Events (CTC-AE) and Late Effects of Normal Tissue, Subjective Objective Management Analytics (LENT-SOMA) (8).

## 2 Timing of radiation therapy

### 2.1 Adjuvant radiation therapy

Improvement in median OS of patients receiving adjuvant chemoradiotherapy was demonstrated in a GITSG (GI Tumor Study Group) study in 1985 (9). and in a 1200-patient retrospective study (10). Both these studies lacked a comparator arm (observation alone is the control arm) and whether the benefit originated from RT or chemotherapy, or both cannot be determined. The EORTC-40891 study compared adjuvant RT with 5-FU to observation in patients with pancreatic and ampullary adenocarcinomas. No statistically significant difference in OS was observed after a median follow up of approximately 12 years (11). The RTOG-9704 study demonstrated no difference in OS between 5-FU or gemcitabine based chemoradiation therapy (CRT) in delivered as adjuvant treatment of resected pancreatic adenocarcinoma. Interestingly, patients with pancreatic head carcinomas demonstrated prolonged OS with gemcitabine-based CRT compared to 5-FU based CRT unlike pancreatic body or tail tumors (12). A secondary analysis of RTOG-9704 study demonstrated an association of radiation quality (defined by adherence to the protocol) may strongly correlate with survival. It was also observed that 48% of the patient population had RT protocol violations which could have led to inferior survival outcomes (13). A prospective evaluation on the predictive ability of postoperative CA 19-9, and margin status on locoregional recurrence and distant metastases was performed. Postoperative Ca 19-9 was significantly associated with locoregional recurrence and distant metastases while margin status was not associated with locoregional recurrence. Interestingly, the results of this study challenged the notion of utility of adjuvant RT based on postoperative margin status (14).

Quality assurance assessment of the treatment plan prior to initiation of radiation treatment for each individual patient is critical. Treatment plans developed by joint efforts of a dosimetrist and radiation oncologist should undergo critical assessment to meet predefined radiation dose constraints to protect organs at risk (OARs) such as small/large bowel, stomach, liver, kidneys, and spinal cord, while ensuring coverage for the clinical and planning target volumes (i.e., CTV and PTV). An institutional weekly peer treatment planning review ensures compliance, through critical peer review of composite dose volume histograms (DVHs), composite isodose distributions for the composite plans in the axial, sagittal and coronal planes at the center of PTV, monitor unit calculations and volume of interest dose statistics.

A subset of patients could benefit from adjuvant RT. RT has been suggested to likely benefit patients with positive resection margins (R1 resection) and or positive lymph nodes. A metanalysis on 4 randomized clinical trials in the adjuvant management of pancreatic adenocarcinoma showed evidence for increased survival benefit with adjuvant CRT in patients with positive resection margins (HR: 0.72; 95% CI: 0.47-1.10) (15). However, the results of this study need to be interpreted cautiously as 2- and 5-year survival rates for R0 and R1 resections were identical. This finding seemed contrary to expectations based upon biologic principles. Similarly, it is unclear whether adjuvant RT would improve survival outcomes in patients with positive post-operative lymph node involvement. In contrast, adjuvant chemoradiotherapy demonstrated improvement in OS compared to adjuvant chemotherapy (HR, 0.9; 95%CI, 0.881-0.977; p=0.004) in patients with resected pancreas adenocarcinoma based on a Surveillance, Epidemiology, and End Results (SEER) database analyses from 2004-2016. This benefit was more pronounced in females and patients with positive lymph node detected on surgical pathology (16).

Prospective randomized studies including stratification factors are key to decipher the benefit of adjuvant RT. Muralidhar, et al. showed a subset of patients with small pancreatic tumors with early nodal metastasis may have a more aggressive cancer biology and could benefit from either early adjuvant therapy or avoiding surgery alone (17). Ma et al. analyzed a total of 7548 patients with stage I to II pancreatic cancer from the National Cancer Database between 2004 to 2015 and illustrated the ideal timing from adjuvant therapy commencement to be between 28 to 59 days after initial surgical resection with significant impact on OS (18).

NRG/RTOG 0848 was a prospectively randomized phase III study performed to answer two questions: whether addition of erlotinib to adjuvant gemcitabine improves primary end point of OS compared to adjuvant gemcitabine alone and whether RT with 5-fluropyrmidine in patients who had not progressed after 5 months of adjuvant chemotherapy (19). There was no significant improvement in OS with the addition of erlotinib to gemcitabine (28.8M vs 29.9M with gemcitabine alone), similar to results generated from CONKO-005 trial. Furthermore, given the improvement in median OS, it was considered that RT would not have adversely impacted the outcomes, pending maturation and final confirmation of the data related to RT.

### 2.2 Neoadjuvant chemoradiotherapy

The therapeutic approach for patients with pancreatic ductal adenocarcinoma (PDAC) is preferably based on a consensus reached from multi-disciplinary discussion. Neoadjuvant approaches have gained traction since they increase the proportion of patients receiving systemic chemotherapy and also provide information on the biology of the cancer before embarking on a major surgical procedure such as a Whipple resection (**Table 1**). Patients receive two to four months of systemic chemotherapy followed by evaluation for response based with an intent to maximize local control, sterilize surgical margins and maximize chances of obtaining an R0 surgical resection.

**Table 1:** List of prospective randomized trials that evaluated the effect of CRT on survival outcomes and adverse effect profile in locally advanced and borderline resectable PDAC (20–26).

Study Title	Setting	Treatment arms	Radio sensitizer and dose of RT	Primary endpoint- results	AE	Comments
LAP-07 (Phase III) N- 449	LAPC (Consolidation CRT)	CRT vs Chemo (after 4 months of Gemcitabine induction chemotherapy)	Capecitabine (800 mg/m2 BID)+RT: 54 Gy in 30 fractions	Median OS - 15.2 M vs 16.5 M	Grade 3/4: Nausea (6% vs 0%)	No significant difference in median OS
FFCD-SFRO (Phase III) N-119	LAPC (Upfront CRT)	CRT (5-FU + cisplatin) -> Gem maintenance vs Gem alone	5-FU: 300 mg/m2/day on day 1-5 for 6 weeks. RT: 60 Gy in 30 fractions	1 year OS – 32% vs 53% (HR:0.54; ci: 0.31-0.96; p=0.06)	Grade 3/4: Induction: 36% vs 22% Maintenance: 32% vs 18%	Study was stopped early as interim analyses showed inferior survival with CRT
ECOG-4201 N-74	LAPC (Upfront CRT)	CRT vs Chemo	Gemcitabine: 600 mg/m2/wk from week 1-5 RT: 50.4 Gy over 5 weeks	Median OS: 11.1 M vs 9.2 M (P-0.17)	Similar Grade 3 toxicity. Higher incidence of grade 4 and 5 toxicities with CRT. (41% vs 9%)	Trial was closed early due to poor accrual. CRT significantly improved median OS.
SCALOP Phase-2 N-114	LAPC (Consolidation CRT)	Gem + capecitabine followed by randomization to Gem based CRT vs capecitabine based CRT	Gem: 300 mg/m2/week Capecitabine: 830 mg/m2 BID on RT days. RT: 50.4 Gy in 28 fractions	9-M PFS: 10.4 M vs 12 M (HR: 0.60, 0.32-1.12; P-0.11)	Grade 3/4 toxicity: Gem group vs Capecitabine group (18% vs 0) (p=0.008)	Capecitabine based CRT is more tolerable and has a trend towards improvement in 9M PFS.
CONKO-007 (Phase III) N-525	LAPC (Consolidation CRT)	Induction chemotherapy followed by CRT vs Chemotherapy alone	Gem: 300 mg/m2/week RT: 50.4 Gy in 28 fractions	Ro resection rate- 25% vs 18%, P-0.1126	Grade 3/4: 73% (CRT) vs 39% (Chemo) (P<0.00001)	R0 resection rate was not statistically significant in the intention to treat population.
PREOPANC (Phase III) N- 246	Resectable and BR PDAC (Upfront CRT)	Neoadjuvant CRT vs upfront surgery	Gemcitabine	OS – 15.7M vs 14.3 M (HR: 0.73; P-0.025) 5-year OS: 20.5% vs 6.5%	52% vs 41% (P-0096)	Although CRT improved OS, whether the benefit is from neoadjuvant gemcitabine vs RT is not clear since control arm went for upfront surgery.
A021501 (Phase II) N-155	BR PDAC (Consolidation CRT)	RT vs Chemo (after 4 months of mFOLFIRINOX induction chemotherapy)	SBRT: 33-40 Gy in 5 fractions HIGRT: 25 Gy in 5 fx	18-M OS rate-47.3% (95%CI: 33.7 – 59.7) vs 67.9% (95%CI: 54.6 – 78.0)		mFOLFIRINOX with hypofractionated RT did not improve OS

LAPC, Locally advanced pancreatic cancer; OS, overall survival; PFS, progression free survival; CRT, chemoradiotherapy; AE, Adverse events; BR, Borderline resectable; PDAC, pancreatic ductal adeno carcinoma; SBRT, stereotactic body RT; HIGRT, Hypofractionated image guided RT; M, month; Gem, gemcitabine.

The PEOPANC study is a randomized phase III trial which studied the OS (primary outcome) benefit of neoadjuvant CRT compared to upfront surgery in resectable and borderline resectable pancreatic cancer patients. Neoadjuvant CRT comprised 3 cycles of gemcitabine combined with 36 Gy RT in 15 fractions followed by surgery and 4 cycles of adjuvant gemcitabine. Patients randomized to the control arm underwent surgery followed by six cycles of adjuvant gemcitabine. The OS was significantly better in neoadjuvant CRT group compared to upfront surgery (15.7 vs 14.3 M, HR: 0.73; P-0.025) after a median follow up of 60 months and this effect was consistent across the subgroups with resectable and borderline resectable pancreatic cancer. The R0 resection rate, the secondary endpoint of the study, with neoadjuvant CRT was 41% compared to 28% in the upfront surgery study arm (P-0.025). Similarly, the percentage of lymph nodes containing tumor cells, tumor size, vascular and perineural invasion were all less frequent in neoadjuvant CRT group (20).

Although this is a positive study, one of the key limitations to its implementation in practice is due to new evidence that adjuvant modified FOLFIRINOX demonstrated superiority over gemcitabine in the PRODIGE-24/CCTG PA.6 trial. It is conceivable that this triplet regimen provides augmented systemic control compared to single agent gemcitabine. Additionally, the criteria used to define resectable and borderline resectable pancreatic cancers in this study are different than those defined in NCCN guidelines. With the recent treatment paradigm shift to neoadjuvant systemic therapy, the relative benefits of gemcitabine-based CRT fare compared to neoadjuvant systemic therapy combinations have become less clear.

Another study, A021501, evaluated the role of SBRT and hypofractionated image guided RT (HIGRT) in patients with borderline resectable PDAC who received RT (33-40 Gy of SBRT or 25 Gy of HIGRT over 5 fractions respectively) or another cycle of systemic chemotherapy after they had completed 7 cycles of neoadjuvant therapy with mFOLFIRINOX. The primary endpoint, 18-month OS rate compared to a historical control of 50% with a pick-the-winner strategy, was not improved with the addition of hypofractionated RT for borderline resectable PDAC. The R0 resection rate, surrogate end point of the study, was 42% with systemic chemotherapy compared to 25% in the RT arm (21). Questions remain as to whether neoadjuvant RT improves the R0 resection rate in the era of systemic chemotherapy with mFOLFIRINOX. Although these findings should be interpreted with caution due to the study limitations including higher percentages of patients in the radiotherapy arm facing chemotherapy dose reductions (60% vs 75%) and treatment delays (49% vs 60%) for unclear reasons. This may explain the paradoxical worsening of R0 rate (88% vs 74%) with local therapy intensification with radiation. Other notable limitations include higher age and lower blood albumin levels in the radiation arm, which were identified as poor prognostic factors in PRODIGE 4/ACCORD 11 trial.

The National Comprehensive Cancer Network (NCCN) guidelines, version 1.2022, recommends consideration of neoadjuvant SBRT as part of clinical trials in high-volume centers for patients who are not candidates for induction chemotherapy, or patients with good performance status and locally advanced disease without systemic metastases. Guidelines also advise against SBRT if direct invasion of bowel or stomach is observed. NCCN panel is awaiting further studies before recommending SBRT as a treatment option for patients with borderline resectable disease, despite safety and feasibility of chemotherapy followed by SBRT in this setting (27).

The NCCN guidelines, version 1.2022, recommends consideration of neoadjuvant SBRT as part of clinical trials in high-volume centers for patients who are not candidates for induction chemotherapy, or patients with good performance status and locally advanced disease without systemic metastases. Guidelines also advise against SBRT if direct invasion of bowel or stomach is observed. The NCCN panel is awaiting further studies before recommending SBRT as a treatment option for patients with borderline resectable disease, despite safety and feasibility of chemotherapy followed by SBRT in this setting.

### 2.3 Locally advanced pancreatic cancer

Radiation therapy in LAPC is mainly used in selected patients who remain progression-free after initial systemic chemotherapy. Despite multiple trials, the role of radiation therapy in unresectable PDAC remains controversial and attempts to definitively prove its value have proven to be elusive. The goal of addition of RT in this setting is to improve surgical outcomes, improve OS and delay local progression. Multiple prospective trials are discussed in this section (**Table 1**), broadly categorized into: those using upfront chemoradiotherapy and those using consolidation chemoradiotherapy. The question of whether CRT improves outcomes for these patients remains to be seen.

#### 2.3.1 Upfront chemoradiotherapy

The role of intensive induction chemoradiotherapy followed by maintenance chemotherapy (gemcitabine) was compared to gemcitabine alone for locally advanced PDAC in a study performed in France (FFCD/SFRO). Cisplatin (days 1-5 during weeks 1 and 5) and 5-Fluorouracil (days 1-5 for 6 weeks) agents were used concurrently with RT (60 Gy over 30 fractions). Overall survival was inferior in the intensive induction therapy arm compared to the gemcitabine alone arm with significantly worse grade 3-4 adverse effects in CRT arm (22).

Radiation therapy with gemcitabine was compared to gemcitabine alone in a randomized study, ECOG-4201. This trial was closed early due to poor accrual. Interestingly, of 74 patients enrolled in the study, concurrent CRT with gemcitabine prolonged median overall survival (mOS) compared to gemcitabine alone (11.1 vs 9.2mo; one sided P: 0.17) with reportedly similar grade 3-4 adverse effect profiles between 2 arms. Median PFS was similar in both arms (23).

Multivariate analysis of the National Cancer Database (NCDB) for 872 patients with primary LAPC treated between 2004–15 showed 134 patients who received SBRT following induction chemotherapy had significant improvement in survival compared to 738 patients who underwent CFRT (HR 0.78, P: 0.25) with mOS of 18.1 versus 15.9 (P= 0.004). This NCDB analysis suggests the benefit of SBRT following induction chemotherapy, which merits further investigation (28).

#### 2.3.2 Consolidation chemoradiotherapy

LAP-07 is an open-label RCT that compared CRT (54 Gy with capecitabine) to chemotherapy in patients with LAPC who were progression-free after four months of systemic therapy. No significant difference in OS or PFS was demonstrated with CRT. CRT seems to have reduced locoregional tumor progression (32% vs. 46%, P-0.04). Interestingly, resection rates were lower in the CRT compared to the chemotherapy arm (3% vs. 6%) (24). The evidence from this study supports that while RT can delay local disease progression, this did not translate into improved survival outcomes.

The SCALOP trial compared the tolerability and safety of gemcitabine-based CRT to capecitabine-based CRT. Although the primary endpoint of the study, progression-free survival, did not meet significance (12 vs. 10.4 M, adjusted HR 0·60, 95% CI 0·32-1·12; p=0·11), median OS was significantly improved in capecitabine arm compared to gemcitabine (15.2 vs. 13.4 M, adjusted hazard ratio [HR] 0·39, 95% CI 0·18-0·81; p=0·012). Capecitabine-based CRT might be a preferable regimen compared to gemcitabine-based CRT for locally advanced pancreatic adenocarcinoma after a course of induction chemotherapy. A significant percentage of patients in the gemcitabine arm developed grade 3-4 hematological toxicities during chemoradiotherapy (18% vs. 0; P-0.008) (25).

CONKO-007 trial is a randomized phase III trial of induction chemotherapy followed by chemoradiotherapy or chemotherapy alone for patients with newly diagnosed non-metastatic LAPC. Patients received induction chemotherapy for 3 months with either FOLFIRINOX or gemcitabine at the treating physician’s discretion (26). Patients who tolerated the induction regimen and those who did not have progression of disease were randomized to CRT with gemcitabine vs chemotherapy alone. Overall survival was the original primary end point which was modified to R0 resection rate due to poor trial accrual. The R0 resection rate was not statistically significant different between study arms in the intention to treat population (25% vs 18%, P-0.1126). In patients who underwent resection, the R0 resection rate (69% vs 50%, P=0.04), CRM negativity (CRM positive: tumor present at the border of resection within 1 mm; 47% vs 25%, P=0.01), and pathologic complete response rates (18% vs 2%, P=0.004) were significantly higher in the CRT compared to the chemotherapy study arms. The median PFS (9 vs 8 mo, P=0.83) or OS (15 vs 15 mo, P=0.71) outcomes were not improved with addition of CRT in this study. Notably, over 90% of patients who achieved surgical resection had FOLFIRINOX therapy which suggests that multi-agent chemotherapy can help convert unresectable PDAC to a resectable state and that RT does not seem to influence the surgical resectability or R0 resection rates. The findings from this study are consistent with prior literature that RT achieves local tumor control in patients with LAPC and that RT may not be enough to drive an OS benefit without systemic disease control.

#### 2.3.3 Intraoperative radiotherapy

A limited number of clinical trials are investigating the role of IORT in pancreatic cancer treatment. A study (NCT01760694) entitled “multi-modality therapy for untreated patients with resectable or marginally resectable pancreatic cancer” from Southwestern Regional Medical Center opened in 2013 to measure the efficacy and safety of IORT in resectable and borderline resectable patients in a multi-modality approach including the use of FOLFIRINOX. The study was terminated in 2014 due to poor recruitment. Another clinical trial from Loyola University (NCT02599662) is looking at adding low kilovoltage IORT in 3 dose tiers, 10Gy, 15Gy, and 20Gy, to establish maximum tolerated dose (MTD) as its primary endpoint. It is also trying to determine the feasibility of IORT, assess acute and chronic side effect profiles and their impacts on patients’ quality of life, and disease-specific outcomes as secondary outcomes. Investigators at Johns Hopkins are conducting a single-arm pilot study (NCT05141513) to assess the safety and feasibility of a single dose of 15Gy of IORT in patients with non-metastatic pancreatic adenocarcinoma (PDAC) who have received neoadjuvant chemotherapy and SBRT and are undergoing surgical resection. Acute and late side effects, as well as disease-specific outcomes, will be investigated. PACER (Pancreatic AdenoCarcinoma with Electron Intraoperative Radiation Therapy) is a phase II multicentric study (NCT03716531) of electron beam intraoperative radiation therapy following chemoradiation in patients with pancreatic cancer and vascular involvement. Outcome measures are the two-year OS, median PFS, local control, and adverse effects.

### 2.4 Latest advances

The use of radiotherapy is evolving, particularly in the neoadjuvant setting, in combination with more effective chemotherapy and newer radiation techniques (**Table 2**). Institutional protocols and expertise guide the methods of radiation delivery, standard versus hypofractionation versus SBRT, and have led to variable practices among radiation oncologists. Learning the current practice patterns, particularly in the neoadjuvant setting, and taking steps towards harmonizing them at high-volume academic centers participating in Canopy Cancer Collective (CCC) is an ongoing initiative. Canopy Cancer Collective is a non-profit organization partnering with leading healthcare systems to create new multi-disciplinary learning networks and improve outcomes for patients and providers with a comprehensive and coordinated effort. A recent survey by Canopy Cancer Collective presented in ASTRO 2022 Annual Meeting of 17 GI experts across the United States showed a significant variation in the use of RT across the stage of disease and treatment parameters like technique, prescription dose, and target volume design. Preoperative measures in resectable or borderline resectable cases included SBRT in 65% of cases and chemoradiation with conventional dose/fractionation versus dose-escalated in 24% vs. 12% of cases, respectively. Definitive measures in LAPC were SBRT, dose-escalated and conventional dose/fractionation chemoradiation in 53%, 41%, and 6% of cases. Future research directions may be influenced by having a better knowledge of the causes of these variations (27).

**Table 2:** Ongoing randomized clinical trials in the Unites States for the use of radiation treatment in the neoadjuvant management of pancreatic cancer.

Study Title, NCT#	Status, phase	Experimental arms	Dosages	Locations
Phase II Study of Stereotactic Body Radiotherapy and Focal Adhesion Kinase Inhibitor in Advanced Pancreas Adenocarcinoma, NCT04331041	Recruiting, phase 2	MR-guided SBRT + Defactinib MR-guided SBRT	MR-guided SBRT of 50 Gy in 5 fractions Defactinib (400 mg) twice a day	Washington University School of Medicine Saint Louis, Missouri
Phase I Study of Concurrent Nab-Paclitaxel + Gemcitabine With Hypofractionated, Ablative Proton Therapy for Locally Advanced Pancreatic Cancer, NCT03652428	Recruiting, phase 1 and 2	Part I: Gemcitabine + nab-paclitaxel: Part II: Hypofractionated ablative pancreatic proton radiation therapy Part III: Surgery, if resectable, then adjuvant chemo per discretion of MD or no further therapy OR Chemo per discretion of MD if not resectable	Pancreatic Proton therapy of 67.5 Gy in 15 fractions Concurrent Gemcitabine + nab-paclitaxel per institutional standard every 7 days for 3 weeks	-MedStar Georgetown University Hospita Washington, District of Columbia-University of Maryland Medical Center/Maryland Proton Treatment Center Baltimore, Maryland
Phase I Study of Precision CRT for Liver-Dominant Metastatic Pancreatic Cancer with Homologous Recombination Deficiency (PreCISeRT), NCT05182112	Recruiting, phase 1	Conformal Radiation Therapy (RT) and Chemotherapy	Whole liver irradiation (WLI) to a total dose 20Gy in 10 fractions Concurrent/adjuvant cisplatin (25 mg/m2) and gemcitabine (600 mg/m2 q2)	Memorial Sloan Kettering Cancer Center and satelites
A Phase I/II Trial of Combination Immunotherapy With Nivolumab and a CCR2/CCR5 Dual Antagonist (BMS-813160) With or Without GVAX Following Chemotherapy and Radiotherapy for Locally Advanced Pancreatic Ductal Adenocarcinomas (PDACs), NCT03767582	Recruiting, phase 1 and 2	Phase I - GVAX/Nivolumab/CCR2/CCR5 dual antagonis Phase II - Arm A: Nivolumab/CCR2/CCR5 dual antagonist Phase II - Arm B: Nivolumab/GVAX/CCR2/CCR5 dual antagonist	Stereotactic Body Radiation (SBRT) SBRT (6.6 Gy over 5 days) Nivolumab (480 mg), day 1 of cycles 1-5 CCR2/CCR5 dual antagonist (150 mg), BID on days 1-28 of cycle 1, then daily on cycles 2-5 GVAX Vaccine (5x10^8 cells) on day 2 of cycles 1-5, six intradermal injections every 4 weeks	Sidney Kimmel Comprehensive Cancer Center Baltimore, Maryland
RT-155: Utilizing Pulsed Low-dose-rate (PLDR) Radiation to Prevent de Novo Stromal Activation; a Neoadjuvant Pancreatic Adenocarcinoma Phase I Trial, NCT04452357	Recruiting, phase 1	PLDR Chemoradiation Dose level 1: 56 Gy Dose level 2: 66 Gy Drug: Gemcitabine	PLDR radiation delivered as 10 fractions of 20 cGy, initiated once every 3 minutes.	Fox Chase Cancer Center Philadelphia, Pennsylvania
An Adaptive Approach to Neoadjuvant Therapy to Maximize Resection Rates for Pancreatic Adenocarcinoma: A Phase II Trial, NCT04594772	Recruiting, phase 2	All patients to receive Neoadjuvant therapy	Radiotherapy *via* a hypofractionated approach over 10 fractions FOLFIRINOX: 5-fluorouracil (2400 mg/m2), irinotecan (180 mg/m2) and oxaliplatin (85 mg/m2) for 2-6 cycles OR gemcitabine (1000 mg/m2) and nab-paclitaxel (125 mg/m2) for 4 cycles	University of Cincinnati Medical Center Cincinnati, Ohio
A Randomized Multicenter Ib/II Study to Assess the Safety & Immunological Effect of Chemoradiation Therapy in Combination with Pembrolizumab Compared to CRT Alone Resectable/Borderline Resectable Pancreatic Cancer, NCT02305186	Recruiting, phase 1 and 2	Neodjuvant CRT + Pembrolizumab Neoadjuvant CRT	Chemoradiation with capecitabine (825 mg/m2 orally twice daily, Monday through Friday, on days of radiation only) and radiation (50.4 Gy in 28 fractions over 28 days) Pembrolizumab (200 mg) IV every 3 weeks on days 1, 22, and 43 concurrent with chemoradiation	-Mayo Clinic Cancer Center, Phoenix, Arizona -Hartford HealthCare, Hartford, Connecticut -University of Miami, Miami, Florida-Dana-Farber Cancer Institute, Boston, Massachusetts-MD Anderson, Houston, Texas-University of Virginia Cancer Center, Charlottesville, Virginia

SBRT requires physics, dosimetry, and therapist support to overcome the technicalities of treatment delivery, including but not limited to image guidance, fiducial marker placements, and respiratory motion control. Image-guided radiation therapy (IGRT) is the delivery of radiation with online imaging and position verification using 2-dimensional kilovoltage (KV) and 3-dimensional cone beam CT (CBCT) images. At the same time, the patient is immobilized on the treatment bed. Fiducial markers are often utilized in hypofractionated treatment delivery techniques, such as SBRT. Their position will be monitored with intra-fraction imaging (IMR) to prevent non-respiratory body motions greater than 3mm. Breath-hold or respiratory gating motion management methods are also crucial to minimize respiration-related motion that impacts treatment delivery to the targets. Gates QA involves verification of respiratory motion measures through a collaboration between a physicist and radiation oncologist.

Parikh and colleagues recently published the findings of a phase II clinical trial on stereotactic MR-guided on-table adaptive radiation therapy (SMART) for patients with borderline or locally advanced pancreatic cancer. 136 patients with biopsy proven adenocarcinoma, who received ≥3 months of chemotherapy and had no evidence of metastatic disease, were enrolled across 13 international sites. Prescribed BED_10_ of 100 Gy was delivered in 5 daily or every other day fractions on a 0.35T MR-^60^Co or MR-linac system with continuous intrafraction cine-MRI, soft tissue tracking, and automatic beam gating. The primary objective was met, through demonstrating <15.8% acute grade 3+ gastrointestinal (GI) toxicity per CTCAE v5.0, in 90 days post treatment (29).

Wisconsin Medical College investigators have just recruited participants for their clinical trial (NCT01918644) investigating neoadjuvant SBRT with concomitant capecitabine in patients with resectable pancreatic cancer. No results are posted yet, but the incidence of dose-limiting toxicities, radiological and pathological response, margin-negative status, and survival outcomes will be measured. Investigators at Massachusetts General Hospital (MGH) reported favorable outcomes with total neoadjuvant therapy in patients with borderline resectable PDAC. Interventions in this phase 2 single-arm clinical trial consisted of FOLFIRINOX for eight cycles followed by individualized chemoradiotherapy (56% short-course, i.e., 5 Gy × 5 with protons and 35% long-course) with capecitabine. More than 70% of patients could complete all chemotherapy cycles. They reported high rates of R0 resection 97% (n = 31) and prolonged PFS of 14.7 months (95% CI, 10.5 mo to not reached) with a 2-year PFS of 55% and a 2-year OS of 72% (30).

Investigators at M.D. Anderson Cancer Center are conducting a phase I clinical trial (NCT04484909) on hafnium oxide nanoparticles NBTXR3 that harbor anti-tumor effects, activated by radiation therapy to improve radiation-induced abscopal effects (31). Investigators are looking for dose-limiting toxicities, maximum tolerated dose, establishing a recommended phase II dose, and evaluating disease-specific outcomes. Patients will receive NBTXR3 intratumorally, then undergo 15 fractions of intensity modulated radiation therapy (IMRT) between days 15-43 without disease progression or unacceptable toxicity.

### 2.5 Selection of patients

Maeda et al. used *in vivo* imaging techniques to investigate the impact of irradiation with a single dose of 4, 12, or 24Gy in pancreatic tumor xenograft models. A single dose of 24 Gy of radiation to the tumors resulted in temporary vascular dysfunction, platelet leucocyte adhesion, and an increase in the expression of HIF-1 alpha. The authors concluded that such biological alterations might affect the tumor’s response to stereotactic body radiation therapy and merit further research (32).

Hu and Guo reviewed synthetic lethality strategies in pancreatic cancer. They suggested that common mutations found in DNA damage repair (DDR) pathways and cell cycle could provide future directions for research (33). An ongoing phase I clinical (NCT01908478) is studying the safety of combining veliparib (ABT-888), a DDR inhibitor with gemcitabine, and intensity modulated radiation therapy in unresectable pancreatic cancer.

Cuneo et al. investigated the combination of another DDR inhibitor, i.e., WEE1 inhibitor AZD1775, with gemcitabine and radiation therapy in 34 patients with unresectable pancreatic cancer in a phase I/II trial (NCT02037230). They determined that the treatment was well tolerated (only 24% of patients developed dose-limiting toxicities like anorexia, nausea, or fatigue) and that the treatment resulted in substantially higher OS (with median OS of 21.7 months and median PFS of 9.4 months) compared to prior results combining gemcitabine and radiation therapy alone (34).

Tomaszewski et al. reported a cohort of 26 patients with borderline resectable and locally advanced PDAC who received Magnetic Resonance Image-guided stereotactic body radiotherapy (MRgRT) of 50 Gy in 5 fractions but did not receive surgery. Delta radiomics analysis of imaging data showed that feature ratios between first and last (5th) fraction correlated with progression-free survival (p = 0.005, HR = 2.75), presenting a potential predictive biomarker for radiation response (35). Rossi et al. studied 71 patients who received induction chemotherapy followed by an ablative dose of radiation in LAPC. They assessed the capability of radiomic features of residual tumor post-induction chemotherapy for predicting resectability. RT regimens included SAbR, 30 Gy in 5 fractions with 50 Gy simultaneous integrated boost (SIB) to the vascular involvement, or with HART, 50.4 Gy in 28 fractions with a vascular SIB of 78.4 Gy. Machine learning algorithms were applied to CT-radiomic features. A model was built to predict surgical resection status and OS with or without surgery, which showed promise but required further validation (36).

## 3 Conclusions

Chemotherapy is the mainstay of treatment for pancreatic ductal adenocarcinoma (PDAC) due to its inherent micrometastatic disease. Neoadjuvant therapeutic strategies have been gaining traction since they increase the proportion of patients receiving systemic chemotherapy and also provide information on the biology of the tumor before embarking on a major surgical procedure such as a Whipple resection. Although it can convert 10-30% of the borderline resectable and locally advanced cases to surgical resection, up to one-third of patients die of complications relating to local progression. Achieving local control in those patients may seem rationale to improve survival outcomes. Since PDAC is inherently radioresistant, a high biologic effective dose is likely needed for effective tumor ablation in the setting of neighboring radiosensitive normal gastrointestinal tissues. Thus, hypofractionation and stereotactic body radiation therapy (SBRT) are gaining more attention, particularly in the neoadjuvant setting. For safe and effective delivery of ablative doses, several important factors need to be considered, including anatomic considerations, dose heterogeneity, organ motion management, and image guidance, as well as the experience of the radiation oncologist, physicist, dosimetrist, and therapist. Multiple ongoing clinical trials are still investigating the role of SBRT, proton therapy, IORT, adaptive therapy, immunotherapy, radiomics, and predictive biomarkers. Widespread implementation of these therapies remains to be achieved as strong peer-reviewed recommendations are hard to establish, and consensus guidelines are under development to support unified practices across the United States.

## Author contributions

MM and FF are co-first authors. MM, FF and SM took part in the conceptualization, writing, editing and revision of the manuscript. HH took part in the conceptualization, editing and revision of the manuscript. NM, SG and RG took part in the editing and revision of the manuscript. All authors contributed to the article and approved the submitted version.
